# Survey results of job status of residents in a standardized residency training program

**DOI:** 10.1186/s12909-019-1718-4

**Published:** 2019-07-25

**Authors:** Yun Zhang, Xiaoming Huang, Hang Li, Xuejun Zeng, Ti Shen

**Affiliations:** 1 Department of General Internal Medicine, Peking Union Medical College Hospital (PUMCH), Chinese Academy of Medical Science (CAMS) and Peking Union Medical College (PUMC), Beijing, 100730 China; 2 Department of Internal Medicine, Peking Union Medical College Hospital (PUMCH), Chinese Academy of Medical Science (CAMS) and Peking Union Medical College (PUMC), Beijing, China

**Keywords:** Standardized residency training program (SRTP), Job burnout, Curriculum setting, Mentoring system

## Abstract

**Background:**

The history of standardized residency training programs (SRTP) in China is not long. As one of the top medical colleges in China, Peking Union Medical College Hospital (PUMCH) has the history and experience of the oldest SRTP in the country. Understanding the job status of PUMCH residents would be conducive to a better development of the national resident training in the future.

**Methods:**

This study analyzed the demographic information, job burnout scale, working time, and job status of postgraduate year 1–3 residents that took part in the SRTP of the Department of Internal Medicine of PUMCH in August 2017.

**Results:**

The survey data of 159 residents (including PUMCH residents, local-resident-trainees, and clinical postgraduates) were collected. The average working time was 11.38 ± 1.55 h per day and 83.28 ± 8.80 h per week. The average night shift frequency was 4.74 ± 0.59 days. There were 100 residents (62.2%) with symptoms of job burnout, which had a certain correlation with working time (*p* < 0.05). The self-evaluation of the clinical postgraduates about their working quality of life was lower than that of other residents (*p* < 0.05). There were various reasons for long working-time, great work pressure, and job burnout. Job burnout was independently associated with the average working time per day (OR = 2.35, 95% CI: 1.47–3.75, *P* < 0.001) and average length of duty period (OR = 1.52, 95% CI: 1.26–1.84, *P* < 0.001).

**Conclusion:**

The job burnout of residents that took part in SRTP at the PUMCH could not be ignored, which had a certain correlation with work time and early training background.

**Electronic supplementary material:**

The online version of this article (10.1186/s12909-019-1718-4) contains supplementary material, which is available to authorized users.

## Background

While the Accreditation Council for Graduate Medical Education (ACGME) has been continuously improving the medical residency training system in the United States for a long time [1–6], China has been progressively promoting the standardized residency training programs (SRTP) for the residents throughout the country over the past ten years. As the top medical college in China, Peking Union Medical College Hospital (PUMCH) has the oldest history and experience of SRTP in the country. In 1921, PUMCH was founded in accordance with the “Johns Hopkins Model”, and it took the lead in advocating and always adhering to formal residency training. Unlike other hospitals in China, which favors medical school graduates to enter a subspecialty directly, the Department of Internal Medicine at PUMCH adheres to the residents’ rotation in all subspecialties of internal medicine, and emphasizes the training of resident’s clinical competence, which is exactly in compliance with the global trend of competency-based medical training.

Job burnout is a common occupational problem in the present society and the incidence of job burnout in healthcare professionals continues to rise [7–9]. Among them, residents take an important part of the daily medical work and tasks. Their job burnout is particularly worrying because of long working time and great pressure of the work [10–13]. Job burnout has a huge impact on the physical and mental health of an individual and in turn on work itself, directly affecting medical safety. Moreover, working time and other factors are clearly related to job burnout [13–15]. A recent study showed that higher preclinical technical knowledge was associated with better clinical competence but higher burnout during clerkships, while higher non-technical characteristics such as morals, social functioning, and physical performance were associated with lower burnout [16]. Core self-evaluation is also a key determinant to adaptation to clinical training, and this self-evaluation varies among individuals [17]. At present, the level of competency training of residents all over the country remains uneven. Nevertheless, all residents all over the country share the same characteristics of bearing tremendous work and learning pressure. There is a lack of studies on the situation and causes of job burnout among residents in China, as well as a lack of concern about the effects of job burnout on their medical behavior. Understanding the work status of PUMCH residents would be conducive to a better development of the national resident training program in the future.

China introduced in 2014 a new policy to integrate the training for clinical postgraduates with SRTP. Therefore, the composition of the population of residents have changed, and now a 3-year SRTP is carried out among the residents employed by PUMCH, local resident trainees employed by other hospitals, and the clinical postgraduates of PUMCH. Different backgrounds, age, medical schools, abilities, working efficiencies, levels of income, and future careers may cause some residents to be under more pressure. Therefore, while paying attention to the medical behavior and ability training of residents, PUMCH also focuses on their physical and mental health. With the improvement of residence training, more attention should be paid on the job status and mental health of the residents, as both of which are associated with the safety and quality of the medical services.

Herein, the present study aimed to investigate the job status of the residents that took part in SRTP at the Department of Internal Medicine in PUMCH, and to find out the reasons for their job burnout, and to explore possible improvement measures.

## Subjects and methods

### Study design

SRTP begins and graduates every September. Therefore, the end of August 2017 was selected for this cross-sectional survey that was carried out by conducting a questionnaire among 159 postgraduate years 1–3 (PGY1–3) residents who took part in SRTP, including the residents employed by PUMCH, local resident trainees employed by other hospitals, and the clinical graduates of PUMCH. In order to protect the privacy of respondents, the anonymous method was used. Questionnaire distribution and recovery were conducted by trained staff. The questionnaire recovery rate was 100% and there were 159 effective questionnaires.

## Methods

The survey was designed specifically for this study and is presented as Supplementary File 1 (English translation). In China, there are both 5-year and 8-year medical schools. Residents are the students who have graduated from medical schools and choose to undergo residency training. Clinical postgraduates are the students who continue their postgraduate studies after they graduated from 5-year medical schools. In recent years, this kind of postgraduate training has been integrated with residency training. The clinical postgraduates have to complete both postgraduate training and residency training at the same time. Students who pass all exams within 3 years after graduation can obtain both postgraduate degree and certificate of residency training. This study included all SRTP trainees admitted by the PUMCH.

1. The demographic information of each subject was collected, including gender (male or female), age, seniority (PGY1–3), source (PUMCH residents, local resident trainees, and clinical postgraduates), and the highest education level before SRTP (bachelor’s degree, master’s degree, and doctoral degree).
2. The Maslach Burnout Inventory-General Survey (MBI-GS) [18] is widely used for the investigation of job burnout in residents all over the world, and its revised Chinese edition (authorized in 2002 by Professor Michael Leiter to Chaoping Li et al., the use of the survey in this study has been approved by the author) has good validity and reliability in China. Indeed, the internal consistency coefficients of the three dimensions are 0.88, 0.83 and 0.82, respectively. The confirmatory factor analysis using AMOS 4.0 showed that GFI, NFI, IFI, and TLI are > 0.90, and RMSEA is 0.08 [19]. There are 16 questions in the survey, involving three dimensions: emotional exhaustion (five questions), cynicism (five questions), and reduced personal accomplishment (six questions). The 7-grade Likert scale is adopted in each question: 0 points for “Never” and 6 points for “Very frequent”. A score < 3 indicates less burnout, 3–5 indicates relatively serious, and > 5 indicates very serious. Reverse scoring is adopted in reduced personal accomplishment. For each dimension, an average score is calculated. Job burnout could be considered if the average score of any dimension is ≥3. Therefore, the subjects were divided into two groups (at least one dimension ≥3 vs. all dimensions < 3).
3. The part of working time consisted of seven questions, which mainly refers to the entries about duty hour by the ACGME Task Force [2], including average working time per day/week, average length of duty period, continuous working time, rate of off duty on time, in-hospital on-call frequency, and night shift cycle. The part of job status consisted of 10 questions, and the 5-point Likert scale is used in the questions involving degree classification, or single choice question, multiple choice question, sequencing question, and open question (Additional file 1). The content included the self-evaluation of job status and self-evaluation of the reasons for job burnout, work pressure, and medical errors. Option setting was in accordance with the results of the studies on the Milestone evaluation system of ACGME and the relevant parts from studies from other countries [20–24]. Open questions were analyzed by content analysis.

### Statistical analysis

This was a convenience sampling of all residents during the study period and no sample size analysis was performed. SPSS 19.0 (IBM, Armonk, NY, USA) was used for statistical analysis. We used standard descriptive statistics and the Fisher exact test or Wilcoxon/two-sample t test, as appropriate, for univariable analyses. Analysis of variance was used for continuous variables. The Pearson’s chi-square test was used for correlation analysis. A multiple logistic regression analysis was performed to identify the factors independently associated with job burnout. A *P*-value < 0.05 was considered statistically significant.

## Results

### Basic information

At the end of August 2017, 159 PGY1–3 residents had participated in the SRTP of the Department of Internal Medicine at PUMCH, including 53 PGY1 residents, 51 PGY2 residents, and 55 PGY3 residents, the basic information of whom was shown in Table 1. All subjects were residents employed by the PUMCH, local resident trainees employed by other hospitals, and the clinical postgraduates of PUMCH. The male to female ratio was 37:122 and the mean age was 27.3 ± 6.2 years. The qualifications of all PUMCH residents were doctoral degrees (92% were graduates of the 8-year clinical medicine program at PUMCH, and they had their internship at PUMCH). The qualifications of all local resident trainees were mainly master’s degrees (accounting for 76%) and the others held doctoral degrees. Clinical postgraduates obtained their bachelor’s degrees and graduated from major medical colleges and universities in China before the SRTP.

**Table 1:** Characteristics of the internal medicine residents

	PGY1	PGY2	PGY3
PUMCH residents	14	13	13
Local resident trainees	3	10	16
Clinical postgraduates	36	28	26
Total	53	51	55

*PGY1–3* Postgraduate year one to three

### Survey results of job burnout

The average score was ≥3 points in at least one dimension for 100 residents (62.2%), indicating that there was a high incidence of job burnout. The scores of all residents in the three dimensions are as follows.

The score of emotional exhaustion (five questions, the maximum possible score for this dimension is 30) was 16.09 ± 7.51, the score of cynicism (five questions, the maximum possible score for this dimension is 30) was 13.22 ± 5.83, and the reverse score of reduced personal accomplishment was 14.06 ± 5.47 (six questions, the maximum possible score for this dimension is 36). There were 48 residents whose average scores of two dimensions were ≥ 3 points and 16 residents had obvious job burnout in all three dimensions. There was small-scale difference in the incidence of job burnout in residents with different seniorities.

### Survey results of working time

Among the 159 residents, the average working time per day was 11.38 ± 1.55 h, the average working time per week was 83.28 ± 8.80 h, the average length of duty period (the continuous working time from the day of duty to the next day) was 32.67 ± 3.44 h, the average rate of off duty on time was 30.51%, the maximum continuous working time was 44.46 ± 7.96 h, the average night shift frequency was 4.74 ± 0.59 days, and the minimum night shift cycle was 3.38 ± 0.75 days.

Table 2 shows that there is no significant difference in working time and night shift frequency among residents with different seniorities, but with an increase in seniority, the rate of off duty time increases. Nevertheless, working time (including average working time per day, average working time per week, and average length of duty period) is positively associated with job burnout. There is no significant association between the rate of off duty time and job burnout.

**Table 2:** Differences in the answers of residents with different seniorities and job burnout

	Seniority				Job burnout		
	PGY1 *N* = 53	PGY2 *N* = 51	PGY3 *N* = 55	*P*	Job burnout *N* = 100	Without job burnout *N* = 59	*p*
Average working time per day (h)	11.64 ± 1.67	11.22 ± 1.43	11.16 ± 1.52	0.324	11.89 ± 1.44	10.41 ± 1.28	< 0.001
Average working time per week (h)	84.79 ± 8.72	82.33 ± 8.89	82.60 ± 8.79	0.365	85.39 ± 8.55	79.63 ± 8.09	0.001
Average length of duty period (h)	32.81 ± 3.53	32.49 ± 3.44	32.72 ± 3.41	0.904	33.79 ± 2.84	30.59 ± 3.53	< 0.001
Rate of off duty on time (%)	24.89 ± 18.87	33.11 ± 19.98	36.40 ± 20.59	0.036	28.42 ± 20.07	34.39 ± 19.75	0.126
Maximum continuous working time (h)	46.34 ± 9.17	43.33 ± 6.88	42.96 ± 6.86	0.110	45.00 ± 8.57	43.46 ± 6.68	0.321
Average night shift frequency (days)	4.72 ± 0.54	4.75 ± 0.61	4.72 ± 0.68	0.958	4.78 ± 0.62	4.66 ± 0.53	0.307
Minimum night shift cycle (days)	3.34 ± 0.73	3.47 ± 0.76	3.28 ± 0.79	0.561	3.37 ± 0.73	3.39 + 0.80	0.882

*PGY1–3* Postgraduate year one to three

**Table 3:** Multivariable analysis to identify the variables related to job burnout

Factor	*p*	Odds ratio	95% confidence interval	
			Upper	Lower
Average working time per day	< 0.001	2.346	1.470	3.747
Average working time per week	0.088	1.067	0.990	1.149
Average length of duty period	< 0.001	1.524	1.263	1.840

A multiple logistic regression analysis identified job burnout as being significantly associated with the average working time per day (OR = 2.35, 95% CI: 1.47–3.75, *P* < 0.001) and with the average length of duty period (OR = 1.52, 95% CI: 1.26–1.84, *P* < 0.001) (Table 3).

### Survey results of job status

#### Self-evaluation of job status (single choice questions)

**Fig. 1:**
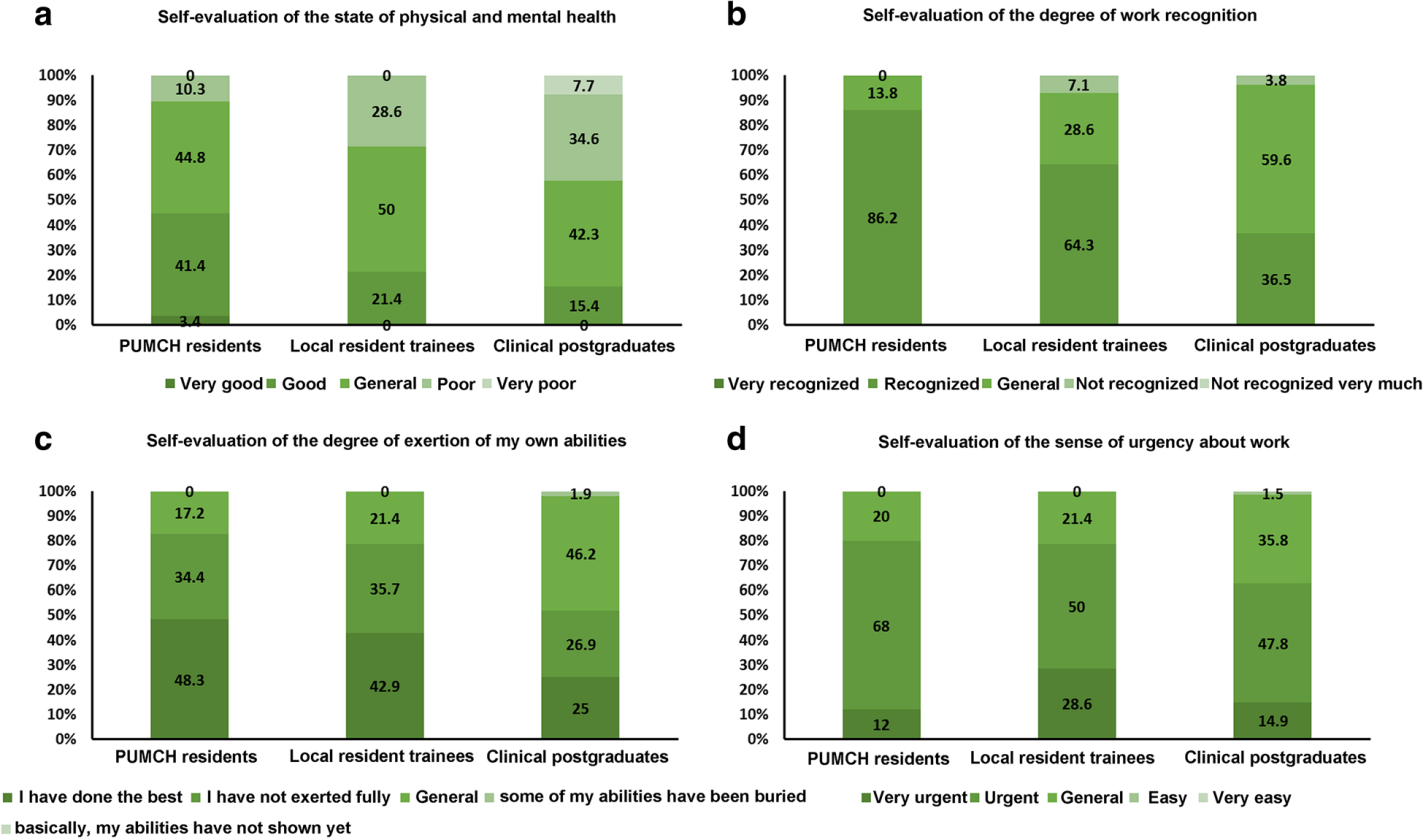
Self-evaluation results of residents with different sources. **a**: Self-evaluation of the state of physical and mental health. **b**: Self-evaluation of the degree of work recognition. **c**: Self-evaluation of the degree of exertion of my own abilities. **d**: Self-evaluation of the sense of urgency about work

The self-evaluation of job status includes four aspects, which are the state of mental health, the degree of work recognition, the degree of the exertion of own abilities, and the sense of urgency about work (Fig. 1). There was a small-scale difference in the results of the four aspects in the self-evaluation of residents with different seniorities. Further classification was carried out according to different sources (PUMCH residents, local resident trainees, and clinical postgraduates). The results of the four aspects of self-evaluation were compared, and they indicated that the self-evaluation of physical and mental health, the degree of work recognition and the degree of the exertion of own abilities of clinical postgraduates were lower than those of the residents who graduated from other medical colleges and universities (*P* < 0.001, P < 0.001, and *P* = 0.008, respectively); no significant difference was observed in the sense of urgency about work (*P* = 0.057).

#### Analysis of the reasons for longer working time (multiple choice questions)

**Fig. 2:**
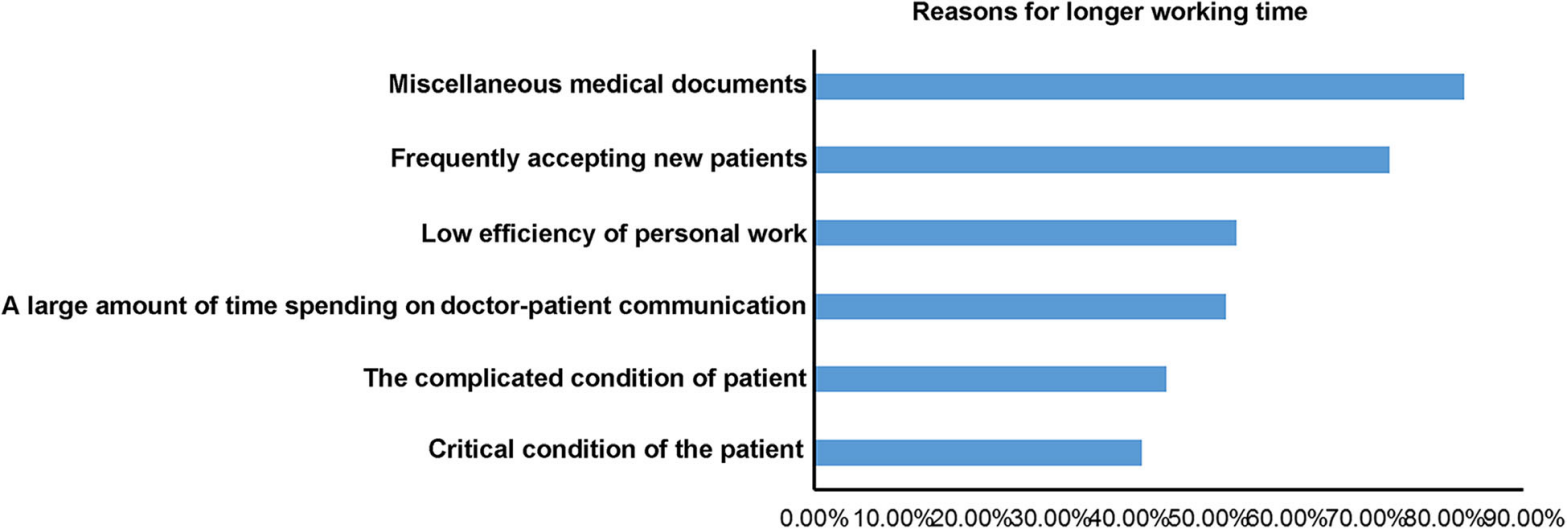
Reasons for longer working time

Among the residents, 82.4 and 72.9% considered that the important reasons for longer working time were miscellaneous medical documents and frequent intake of new patients, respectively, followed by the low efficiency of personal work, a large amount of time spent on doctor-patient communication, and the complicated and critical condition of the patient. See Fig. 2 for details.

#### Medical errors in clinical practice (multiple choice questions)

Among the residents, 10.1% thought that they never had an obvious medical error, while 67.3% forgot to have examinations or medicine prescribed to patients, 62.3% wrote the doctor’s advice wrongly, 45.3% failed to complete medical documents in time, 34.0% were inaccurate to judge patient’s condition when they were on duty, and 31.5% were mentally blocked or fell asleep, leading to inefficiency in following the important instructions of a superior physician while making ward rounds. Moreover, 6.9% made a mistake while drawing blood from patients for testing.

#### Work pressure sources (multiple choice questions)

**Fig. 3:**
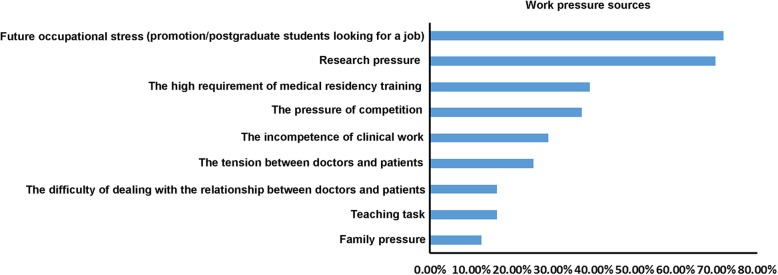
Work pressure sources

The numbers of residents who chose future occupational stress (deciding which specialized department to work/promotion/postgraduate students looking for job) and research pressure as their answers in self-evaluation, were the largest, with 71.7 and 69.8%, respectively. The top five answers also included the high requirement of medical residency training, the pressure of competition, and the incompetence of clinical work (Fig. 3).

#### Self-evaluation of job burnout (multiple choice questions)

**Fig. 4:**
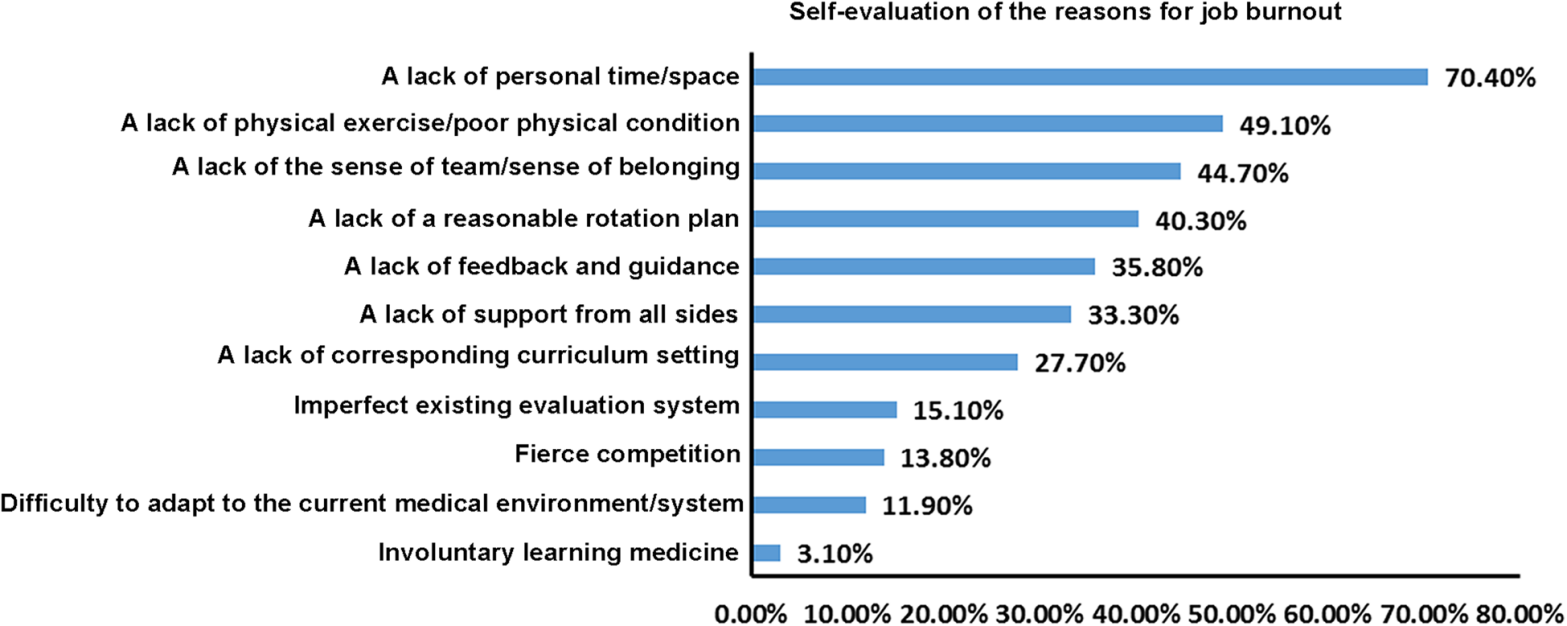
Self-evaluation of the reasons for job burnout

Among the residents, 86.8% believed that they had a certain degree of job burnout. The top five specific reasons included a lack of personal time/space, a lack of physical exercise/poor physical condition, a lack of the sense of team/sense of belonging, a lack of a reasonable rotation plan, and a lack of feedback and guidance (Fig. 4).

#### Impressively negative life/work of residents

Among the residents, 37.7% expressed that they have experienced significant negative life/work issues; 20% of them did not want to share these issues, while 80% of them shared these issues with us with very short descriptive statements. The issues were mainly focused on the following aspects: medical errors (35.4%), unsuccessful case of doctor-patient communication (16.7%), not recognized by superior or colleague (14.6%), medical error made by myself (14.6%), failure to treat patients successfully (12.5%), and neglected the family (6.3%).

#### Measures for individuals obtaining the most benefit (single choice questions)

The answers of the residents were Handoff system (after night shift, work can be handed over to specified colleagues) (26.4%), advance planning of rotation arrangement (24.5%), a specialized superior physician/tutor providing a full range of help (back-up) (15.7%), setting a more reasonable and individualized goal (12.6%), initiating related courses (such as relieving pressure and improving efficiency) (8.8%), organizing holidays (6.9%), increasing income (3.8%), and others (1.3%).

## Discussion

With the wider implementation of the SRTP, more and more subjects such as postgraduates are being included in the SRTP. The capabilities of such subjects are lower than that of the graduates after 8 years of medical education. In addition, some of the subjects still need to attend class and study during residency, which could increase work time and decrease work efficiency. The SRTP itself is not associated with job burnout, while different backgrounds, age, medical schools, abilities, working efficiencies, levels of income, and future careers may cause some residents to be under more pressure. Therefore, job burnout could appear after the subjects are in such state for relatively long time. Nevertheless, the exact reasons and factors leading to job burnout among Chinese residents in the SRTP are poorly understood. The present was undertaken with the aim of ultimately improving residence training by paying more attention to the job status and mental health of the residents. This should be conducive to improved safety and quality of the provided medical services.

Psychologists believe that job burnout is a slow development process and it can exist in the form of a long-term stress beginning at an early stage [14], similar to the work of residents. Job burnout is closely associated with depression and studies from various countries indicate that the proportion of job burnout in residents is as high as 50–76% [11, 12, 20–23, 25–28]. The incidence of depression and other affective disorders in residents is even higher than that of the general population [11, 20–23, 25, 29], but the current level of concern about it in China is far from being enough. As a top medical college in China, Peking Union Medical College (PUMC) and PUMCH are going to integrate their nearly 100 years of experience with the opportunities and challenges at the new era and contribute to medical residency training in China. This study was the first formal survey of all residents taking part in the 3-year SRTP from the Department of Internal Medicine of PUMCH in China. The results showed that among PGY1–3 residents, 62.2% had symptoms of job burnout, which is consistent with the previous studies [11, 12, 20–23, 25–28], and indicating that it is a matter demanding urgency to take appropriate measures to improve the physical and mental health of residents.

China has a huge population and the shortage of doctors is a very serious social issue. Chinese residents who are just entering the medical profession will face a higher pressure and work burden than those in western countries. In addition, because of cultural differences, Chinese people are often not very good at sharing their inner feelings with others. Therefore, direct application of western research results and experience to Chinese young doctors may not have the same effect. Additional studies are necessary to determine the characteristics of the Chinese residents and appropriate interventions. In this study, the sources of stress among residents at the Department of Internal Medicine in PUMCH were investigated. More than 2/3 residents had the pressure of future career development and the pressure of scientific research, the high requirement of SRTP, and tension in the relationship between doctors and patients. Relatively speaking, there is little pressure from teaching and family. The data of this study was only from the single center of PUMCH, and a certain single-center bias might exist. For example, the doctors at PUMCH are well qualified for clinical and teaching work, in comparison with other medical colleges, the competition is more intense, and the self-requirement is higher.

### Job burnout may eventually affect medical safety

Medical knowledge and the performance of clinical work are often used as the main indicators to evaluate the competency of residents, but their physical and mental health would also affect their training and, in turn, affect medical safety [29]. “Medical errors” during the clinical work of residents was investigated in this study and the results showed that 89.9% of the residents admitted having made “medical errors” at some point. Although the association between job burnout and “medical errors” was not directly investigated, previous studies showed that the degree of job burnout and the degree of depression were significantly related to the occurrence of medical errors [10, 12, 20, 30]. A lack of sleep and clinical experience, high work intensity, lack of proper supervision, and non-standard shift handover are all related to medical errors in residents. Moreover, these factors are also considered as the common causes of job burnout in residents [31]. Therefore, in addition to the corresponding direct improvement measures for these medical errors, it is urgent to pay attention to and take measures to mitigate the emotional barriers of residents such as job burnout, anxiety, and depression.

### Significance of limiting working time to job burnout

The risk factors for job burnout include gender, educational level, the nature of the work (workload and stress), and income. Previous studies found that males had a higher tendency for cynicism, while females had higher levels of emotional exhaustion [11, 32]. In this study, the proportion of male residents was low. Therefore, the study group showed more important emotional exhaustion dimension of job burnout. The results of some studies on the job burnout of residents suggest that in addition to work stress, job burnout is also related to the economic situation, life pressure, and training assessment. At the same time, working time is associated with job burnout, insomnia, and depression. Frequent night shifts, increased workload on duty, and off night shift late would cause job burnout and further reduce the opportunities to participate in medical training activities [10, 23, 33]. Being resident is the first job for medical students after their graduation from medical colleges and the little working experience might lead to low work efficiency. Moreover, PUMCH is a diagnosis and treatment center for difficult and severe diseases in China, the proportion of critical and difficult cases in each ward of the department of internal medicine accounts for nearly half of the beds, which is a great challenge for residents with low seniority. The previous questionnaire survey from our group showed that overtime is very common in the residents of the department of internal medicine at PUMCH [34]. The results of this study also confirmed that working time is positively associated with job burnout. In the self-analysis of the cause of job burnout, a lack of personal time/space was chosen by a dominant number of residents. A study found that reducing duty frequency and working time is beneficial to reduce medical errors in ICU residents [35]. Therefore, in order to improve health care quality and to ensure medical safety, the ACGME had made a clear limitation on the duration of working time for residents in the requirements of medical residency training program in 2003 and 2011, respectively [2], which included that maximum working time to be no more than 80 h per week, maximum duration of duty period to be no more than 30 h (24 h on the day of duty, no more than 6 h on the next day), and the in-hospital on-call frequency was no less than 3 days. The 2011 update version required the continuous working time of interns to be no more than 16 h, and the continuous working time for residents with high seniority to be no more than 28 h (24 h on the day of duty+ 4 h on the next day), which were all shorter than the working time of the residents in the Department of Internal Medicine at PUMCH.

### Exploring real effective measures for improvement

The proportion of job burnout has been decreased by limiting the length of working time, but new problems arose that require attention. Studies showed that after DHRs, the attendance rate of residents in teaching activities declined (the reduced length of working hours was from the reduced degree of participation in the course, and the efficiency of clinical work was not improved) and the effects of SRTP were questioned by faculties, patients, and even trainees themselves. Studies suggested that the limitation of the length of working time was not good for medical safety and medical residency training. For example, in the first two years after the promulgation of DHRs, the mortality rates of patients in many teaching hospitals were improved, indicating that the improvement of medical quality by this measure was very limited [5, 36, 37]. Some studies suggested that the job burnout of residents did not depict significant improvement in spite of the limitation of the duration of working time [38, 39].

Through the present study, there were many reasons for the longer working time of residents, including miscellaneous medical documents, frequent intake of new patients, low efficiency of personal work, a large amount of time spending on doctor-patient communication, and complicated and critical condition of the patient. Therefore, the improvement in working time alone could not effectively solve the root cause of job burnout in residents. The practical and meaningful measures should include more reasonable arrangement of clinical work, curriculum setting, and professional quality training in SRTP, in order to ensure medical safety and reduce the incidence of job burnout in residents [10]. Based on this survey and the literature, some improvement measures are possible, as below.

1. Off night shift system (handover, transition): Off night shift/handover system could guarantee the length of continuous working time in residents, but shortening the length of working time increases the frequency of handover to some extent. Many studies showed that handover is often detrimental to medical safety, the reasons include nonstandard handover and a lack of corresponding courses in medical education [24, 40]. Therefore, how effectively and safely to carry out handover becomes the next problem to be solved. At present, many countries have begun to set up courses about handover training, but it is still incomplete in SRTP in China.
2. Mentoring system: Mentoring system or establishing a more intimate relationship between teachers and residents could help set up a more reasonable and individualized target and coordinate rotation plan, which is beneficial to the growth as well as the physical and mental health of residents in medical teaching and research [41–43].
3. Curriculum setting should integrate resources, attention to be paid to teaching students in accordance with their aptitude and demand, and carry out stratified teaching. The training of professional accomplishment, communication skills, balancing work and family, healthy diet and exercise, and improvement of work efficiency should be introduced into the SRTP or even in the education system of medical colleges [6]. It is very difficult to improve job burnout once it arises [39]. This study showed that there is little difference between working time and the incidence of job burnout in PGY1–3 residents, and the incidence of job burnout in most of the residents who graduated from PUMC is relatively lower. Their self-evaluation of physical and mental health, the degree of work recognition and the degree of exertion of their own abilities are superior to those of the residents who graduated from other medical colleges and universities. The reason might be the cultivation of correct professionalism and physical and mental health by the PUMC during the training of medical students, which is also consistent with previous studies from other countries [11, 12, 20–23, 25–28]. Measures to prevent job burnout should be carried out as soon as possible, and it is more effective if they are carried out during the training in medical colleges.

The present study is not without limitations. The sample size was small and from a single hospital, but including different hospitals with different training programs and workload would lead to bias. The proportions of female and male residents were unequal, preventing formal subgroup analyses. Finally, Chinese residents and those from other countries probably have different cultural confounders, modifiers, and determinants, but additional study would be necessary to determine them.

## Conclusions

The purpose of the SRTP is to improve the overall medical standard in China, but at present, the job status and job burnout of residents cannot be ignored. In order to ensure the safety of medical service, more attention should be paid to the physical and mental health of the residents and the improvement of their competence and professionalism. The present preliminary investigation provides a basis for the improvement of the SRTP in China.

## Additional file

Additional file 1: Work status questionnaire of residents in Peking Union Medical College Hospital. (DOCX 36 kb)

## Data Availability

The datasets used and/or analyzed during the current study are available from the corresponding author on reasonable request.

## Abbreviations

ACGME: Accreditation Council for Graduate Medical Education
MBI-GS: Maslach Burnout Inventory-General Survey
PGY: postgraduate year
PUMC: Peking Union Medical College
PUMCH: Peking Union Medical College Hospital
SRTP: standardized residency training programs
