# Supplementary material for: Survey results of job status of residents in a standardized residency training program
Source: BMC Med Educ. 2019 Jul 25;19:281. doi: 10.1186/s12909-019-1718-4 (PMC6659202; doi:10.1186/s12909-019-1718-4)
Supplement: Supplementary file 1 — Work status questionnaire of residents in Peking Union Medical College Hospital. (DOCX 36 kb) [file 12909_2019_1718_MOESM1_ESM.docx]

**Work status questionnaire of residents in Peking Union Medical College Hospital**

1. **Basic information**

1) Gender: ① Male; ② Female.

2) Age: __________ years old.

3) Seniority: ① Graduate student in 2015; ② Graduate student in 2016; ③ Resident in 2015; ④ Resident in 2016.

4) The highest education level obtained: ① Bachelor’s degree; ② Master’s degree; ③ Doctoral degree.

5) Personal information: ① Unmarried (single); ② Unmarried (non-single); ③ Married.

6) Your average monthly income: ① <2000 RMB; ② 2000-3999 RMB; ③ 4000-5999 RMB; ④ 6000-7999 RMB; ⑤ >8000 RMB.

7) Would you be more willing to fill out the questionnaire if we use a mobile/web/WeChat version?

① Yes ② No

This questionnaire is designed to understand the working status of residents (including psychological status, working hours, etc.) with different years of experience. We guarantee that all the collected information will be used for research purposes only. Your personal information will be kept confidential. Thank you very much for taking time off your busy schedule to complete our questionnaire! We hope that our questionnaire will be able to strive for more benefits for residents!

If you have any question, please feel free to talk to our staff distributing the questionnaire!

**2 Job burnout scale**

A total of 16 items are described below. Please determine how often they happen in your hospital or in your own situation according to your feelings and experience. If you have never had this idea or experience, please choose 0. If you have had this idea or experience, please choose the right number.

| 0 | 1 | 2 | 3 | 4 | 5 | 6 |
| --- | --- | --- | --- | --- | --- | --- |
| Never | Rare  Several times a year or less | Occasionally  Once a month or less | Often  Several time a month | Frequently  once a week | Very frequently  Several times a week | Every day |

| 1 | The work makes me feel physically and mentally exhausted | 0 | 1 | 2 | 3 | 4 | 5 | 6 |
| --- | --- | --- | --- | --- | --- | --- | --- | --- |
| 2 | I feel exhausted when I get off work | 0 | 1 | 2 | 3 | 4 | 5 | 6 |
| 3 | I feel very tired when I get up in the morning thinking that I have to face a whole day of work | 0 | 1 | 2 | 3 | 4 | 5 | 6 |
| 4 | It is really stressful for me to have a whole day of work | 0 | 1 | 2 | 3 | 4 | 5 | 6 |
| 5 | I can effectively solve problems in my work | 0 | 1 | 2 | 3 | 4 | 5 | 6 |
| 6 | The work makes me feel like I’m going to collapse | 0 | 1 | 2 | 3 | 4 | 5 | 6 |
| 7 | I feel that I am making useful contribution to the hospital | 0 | 1 | 2 | 3 | 4 | 5 | 6 |
| 8 | I have become less and less interested in my work since I started this job | 0 | 1 | 2 | 3 | 4 | 5 | 6 |
| 9 | I am not as enthusiastic about my work as I used to be. | 0 | 1 | 2 | 3 | 4 | 5 | 6 |
| 10 | I my opinion, I am good at my work | 0 | 1 | 2 | 3 | 4 | 5 | 6 |
| 11 | I am very happy when I finish some things at work | 0 | 1 | 2 | 3 | 4 | 5 | 6 |
| 12 | I complete a lot of valuable work | 0 | 1 | 2 | 3 | 4 | 5 | 6 |
| 13 | I only want to do my job, but I don’t want to be disturbed by other things. | 0 | 1 | 2 | 3 | 4 | 5 | 6 |
| 14 | I doubt the meaning of the work I am doing. | 0 | 1 | 2 | 3 | 4 | 5 | 6 |
| 15 | I have become less and less interested in whether the work I am doing is contributory | 0 | 1 | 2 | 3 | 4 | 5 | 6 |
| 16 | I believe I can accomplish all the work effectively | 0 | 1 | 2 | 3 | 4 | 5 | 6 |

**3. Working hours (evaluated within the past 12 months)**

1) How many hours do you work on average per day?

A. <8 hours; B. 8-10 hours; C. 10-12 hours; D. 12-14 hours; E. >14 hours.

2) How many hours do you work on average per week (routine work + on-duty work)?

A. 60-70 hours; B. 70-80 hours; C. 80-90 hours; D. 90-100 hours; E. >100 hours.

3) How many continuous working hours do you do on your on-duty day and on the next working day?

A. <24 hours; B. 24-28 hours; C. 28-32 hours; D. 32-36 hours; E. >36 hours.

4) Rate of off-duty time (for example, the latest off-duty time is before 18:30 pm on the day you are not on-duty) __________

A. <20%; B. 20-40%; C.40-60%; D. 60-80%; E. 80-100%.

5) Was it the longest record of continuous working time in the ward/hospital?

A. <24 hours; B. 24-36 hours; C. 36-48 hours; D. 48-60 hours; E. >60 hours.

6) The average cycle of your night shift is __________

A. 3 days; B. 4 days; C. 5 days; D. 6 days; E. >7 days.

7) The shortest cycle of night shift you experienced is __________

A. 2 days; B. 3 days; C. 4 days; D. 5 days; E. >6 days.

**4. Working status (within the past 12 months).**

1) What do you think of your physical and mental health?

A. Very good; B. Good; C. General; D. Poor; E. Very poor.

2) Do you think that your work has been recognized by your leaders and colleagues?

A. Very recognized; B. Recognized; C. General; D. Not recognized; E. Not recognized very much.

3) Do you think that your ability has been fully exerted?

A. I have done my best; B. I have not exerted fully; C. General; D. Some of my abilities have been buried; E. Basically, my abilities have not shown yet.

4) How do you feel about the urgency of your work?

A. Very urgent; B. Urgent; C. General; D. Easy; E. Very easy.

5) What do you think are the reasons that would lead to longer working time, in your specific case (multiple choice and in order) _____________

A. Frequently accepting new patients; B. a large amount of time spent on doctor-patient communication; C. Critically ill patients; D. complicated and critical condition of the patient; E. Low efficiency of personal work; F. a large amount of time spending on various levels of ward rounds; G. Miscellaneous medical documents; H. Other

6) What are the sources of psychological stress in your daily work (multiple choice and in order) ____________________

A. The high requirements of medical residency training; B. The tension between doctors and patients; C. The difficulty of dealing with the relationship between doctors and nurses; D. Research pressure; E. Future occupation stress (looking for work/Chief on duty/specialization); F. Teaching task; G. Family pressure; H. The incompetence in clinical work; I. The pressure of competition; J. Other.

7) Have you ever experienced “medical errors” in your clinical work?

If you have, which of the following situations have you experienced__________________

1. Have written doctor’s advice wrongly; B. Have forgotten to prescribe examinations or medicine to patients; C. Had been inaccurate to judge patient's condition when on duty; D. Failed to complete medical documents in time; E. Have been mentally blocked or have fallen asleep; F. Other.
2. If you have not experienced medical errors, please go directly to question 8.

8) Have you ever experienced impressively negative life/work issues? __________

A. No

B. If you have experience, can you share it with us:

___________________________________________________________________

___________________________________________________________________

9) Are you feeling that you are always not interested in your work? Are you full of boredom with your current professional status? Are you experiencing significant reduction in work performance and efficiency, and feeling physically exhausted, but you used to be extremely efficient? If you have the above “symptoms”, you are probably facing a crisis of professional burnout. What do you think are the reasons leading to your “professional burnout” (multiple choice)? _________________

A. Lack of feedback and guidance; B. Lack of corresponding curriculum setting; C. Lack of sense of team /sense of belonging; D. Lack of supports from all sides; E. Lack of reasonable rotation plan; F. Fierce competition; G. Lack of personal time/space; H. Difficult to adapt to the medical environment/system; I. Imperfect existing evaluation system; J. Lack of physical exercise/poor health condition; K. Involuntary learning medicine; L. Other.

If you do not have the above symptoms, please go to question 10.

10) Which of the following measures do you need most to help you (single choice)

A. A specialized superior physician/tutor providing a full range of help (back-up);

B. Initiating related courses (such as relieving pressure and improving efficiency, etc.);

C. Handover system (after night shift, work can be handed over to specified colleagues);

D. Setting a more reasonable and individualized goal;

E. Advance planning of rotation arrangement;

F. Other.

**5. Summary**

The overall score of your work, life and learning status in the past 12 months.

(Full mark: 10 points; minimum: 1 point; maximum: 10 points)

1. Score of overall quality of life:____________, your expectation:____________.
2. Score of overall quality of work:____________, your expectation: ____________.
3. Score of the quality of patient treatment:____________, your expectation:____________.
4. Score of teaching/learning quality:___________, your expectation:____________.
5. Score of fatigue (10 points is the most fatigued):____________, your expectation:____________.

In addition, do you want to share your experience with us, or do you have any suggestion?

____________________________________________________________________________________________________________________________________________________________________________________________________________________________________________________________________________________
